# The use of social media after bereavement by suicide: results from a French online survey

**DOI:** 10.1186/s12888-024-05761-9

**Published:** 2024-04-23

**Authors:** Edouard Leaune, Héloïse Rouzé, Laurène Lestienne, Kushtrim Bislimi, Margot Morgiève, Benoit Chalancon, Pauline Lau-Taï, Guillaume Vaiva, Pierre Grandgenèvre, Julie Haesebaert, Emmanuel Poulet

**Affiliations:** 1 Research on Healthcare Performance RESHAPE, INSERM U1290, Université Claude Bernard Lyon 1, Lyon, France; 2 Center for Suicide Prevention, Centre Hospitalier le Vinatier, 95, Boulevard Pinel, 69500 BRON, France; 3 Groupement d’Etude et de Prévention du Suicide, Brest, France; 4 Service Recherche et Epidémiologie Cliniques, Pôle Santé Publique, Hospices Civils de Lyon, Lyon, France; 5 Cermes3, Université Paris Cité, CNRS, Inserm, Paris, France; 6 U1172-LilNCog-Lille Neuroscience & Cognition, University of Lille, CHU Lille, Inserm, Lille, France; 7 Centre National de Ressources & Résilience Pour Les psychotraumatismes (Cn2r Lille Paris), Lille, France; 8 Department of Emergency Psychiatry, Hospital Edouard Herriot, Hospices Civils de Lyon, Lyon, France

**Keywords:** Suicide bereavement, Social media, Suicide, Grief

## Abstract

**Background:**

According to recent research, the Internet and social media are shaping and changing how we die and mourn. However, the use of social media after bereavement by suicide remains poorly understood. Thus, emerging research is needed to better assess the role that social media can play after bereavement by suicide. The objective of our study was to evaluate the use of social media in French people bereaved by suicide and to assess their expectations toward social media.

**Method:**

We conducted a national cross-sectional online survey including French people bereaved by suicide assessing their use of social media after the death of their relative. All adults bereaved by suicide were eligible to participate in the study. An online 26-item questionnaire collected sociodemographic and loss-related characteristics and evaluated four dimensions: (1) the use of social media in daily life, (2) the perceived needs regarding suicide bereavement, (3) the use of social media associated with the suicide loss, and (4) the expectations regarding the development of an online resource for people bereaved by suicide and proposals regarding the development of such a resource.

**Results:**

Among 401 participants, 61.6% reported using social media after the death of their relative by suicide, especially those recently bereaved, those receiving counseling and bereaved parents. The participants mainly used social media to reach peers bereaved by suicide and to memorialize, while they expected social media to help them finding information on suicide and accessing bereaved peers. Younger participants were more prone to use social media to memorialize, while bereaved partners and those bereaved by the suicide of a parent were less prone to use them with such aim.

**Discussion:**

A large part of people bereaved by suicide use social media for their grief process, mainly to contact peers bereaved by suicide and to memorialize their loved one. According to or results, social media contributes to contemporary grief processes after suicide bereavement and can be seen as putative means to improve the well-being of people bereaved by suicide.

**Supplementary Information:**

The online version contains supplementary material available at 10.1186/s12888-024-05761-9.

## Background

According to recent research, the Internet and social media are shaping and changing “how we die and mourn” [1, 2]. For example, Walter et al. [1] demonstrated through a literature review that Internet is used for online practices in relation to dying, including online support groups for people with life threatening diseases, digital inclusion of those nearing the end of life or online facilities during funerals, but also in relation to bereavement. Moreover, in 2012, nearly 3 million users’ profiles were maintained on Facebook as memorials [3], demonstrating the emergence of new approaches to bereavement and memorialization, which is the act of preserving memories, events or people coming in many forms ranging from ceremonies to gatherings to physical pieces of remembrance, such as a memorial, urn or both [4]. Social media is a form of digital technology that enables the sharing of ideas and information, including text and visuals, through virtual networks and communities [5]. It is emerging as a new way for people bereaved by suicide to grieve and mourn [6].

Several studies have assessed how social media can help people who are bereaved remembering their deceased loved one [7, 8]. These studies have reported innovative digital behaviors such as continuing bonds online or the expression of emotions and feelings through online platforms. Continuing bonds has been defined as ‘‘the presence of an ongoing inner relationship with the deceased person by the bereaved individual’’ [9] and has been showed to be associated with positive experiences in most people bereaved by suicide. In a recent systematic review [10], women bereaved by suicide were reported to create more online memorials than males and, conversely, men appeared to engage more in avoidant coping strategies and would express their grief less openly. Additionally, social media are used by people who are bereaved to create online communities where they can share their experiences, emotions, and feelings about the death of their relative and their bereavement process. According to the *social media mourning model* developed by Moore et al. [8], social networking sites are used to share information with family or friends, discuss death with other mourners and with a broader mourning community and commemorate and continue connection to the deceased.

According to a recent meta-analysis, those exposed to suicide in the past year represent approximately 4% of the population and the lifetime prevalence of suicide exposure in the family is estimated to be 3.8% in the general population [11]. Suicide bereavement is highly distressing for mental and physical health, with a higher prevalence of suicidal behaviors, prolonged grief disorder and mood disorders than other causes of grief [12, 13]. Social stigma is particularly prevalent for people bereaved by suicide and can lead to social withdrawal and loneliness [14, 15]. It may also lead to a lack of access to health care or psychological support. In this context, digital resources have been identified as a source of nonjudgmental support around the clock to people bereaved by suicide. According to a recent systematic review, digital resources may help to overcome barriers to access to resources and support because people bereaved by suicide may feel less stigmatized on the internet and are free to disconnect from the virtual world at any time [16].

However, the use of social media after suicide bereavement remains poorly understood. In a cross-sectional study [17], Bailey et al. found that 222 Australian users of Internet forums about suicide bereavement reported that their participation in the forums was beneficial. Surprisingly, no other study has evaluated how people bereaved by suicide use social media, why they participate in online forums or what they expect from social media after the death of their relative by suicide. Thus, emerging research is needed to better assess the role that social media can play after bereavement by suicide. For example, there is a need to better understand how social media are used by people bereaved by suicide depending on their age, gender or relationship to their loved one who has died by suicide. Based on previous research, it is possible to hypothesize that individuals who have lost a child to suicide, as well as women and young adults, may exhibit distinct social media usage patterns following such a loss [4, 6–8]. For example, a recent systematic review found differences in the use of online memorials after bereavement between men and women [10], while a study of Australian youth reported that boys and girls had similar associations between social media use, body image concerns, and eating disorders [18]. Previous studies have also reported contrasting results regarding the use of social media for grief concerns in youths, with some reporting greater use and others reporting lower use depending on the samples [19–21]. The objective of our study was to evaluate the use of social media in French people bereaved by suicide and to assess their expectations toward social media.

## Methods

### Data collection

This online survey is part of the ESPOIR_2_S study, a mixed-method collaborative and participatory user-centered study that aims to build resources from the perspectives of people bereaved by suicide through the iterative process of the *Information System Research (ISR) framework*. The ISR framework employs various design processes to build a product or design an artifact, such as a mental health online resource [22, 23]. The protocol of the study has been exhaustively described elsewhere [24, 25]. In this article, we describe the results of the first quantitative stage of the study, namely, the *relevance cycle*, which aimed to collect the use, needs and expectations of people bereaved by suicide regarding the internet and social media. The *Checklist for Reporting Results of Internet E-Survey* (CHERRIES) was used to guide our reporting of the results [26].

We conducted a national cross-sectional online survey in France from July to October 2021. The data were collected through a 26-item online questionnaire using single and multiple choice closed-ended questions (Supplementary Material 1), which was built by our pluri-professional research team (psychiatrists, psychologists, public health researchers, sociologists, people with lived experience) for the study according to a recent systematic review of online resources for suicide bereavement [16]. It was hosted on the website Lime Survey. This nonvalidated questionnaire collected six sociodemographic and loss-related characteristics (age, gender, relation to the deceased, time since loss, access to counseling and type of counseling) and evaluated four dimensions: (1) three single choice closed-ended questions on the use of social media in daily life (frequency of use of Internet and social media, type of digital resource(s) used), (2) one single choice closed-ended question using a Likert scale from “not at all” to “extremely” on perceived needs regarding suicide bereavement (social support, professional counseling, peer support, meaning-making), (3) nine single and multiple choice closed-ended questions on the use of social media associated with the suicide loss (frequency of use, type(s) of resources used, reasons for using online resources, satisfaction with the resources), and (4) seven single and multiple choice closed-ended questions on expectations regarding the development of an online resource for people bereaved by suicide (type of resource, technology, typology of use) and proposals regarding the development of such a resource. No scores were calculated through the questionnaire. The results on the use of the Internet have been published elsewhere [24].

### Participants and recruitment

All adults bereaved by suicide were eligible to participate in the study. As we aimed to evaluate the use of social media throughout the bereavement process, we did not use any exclusion criteria based on the time since the loss. Because the questionnaire was in French, being able to read and write in French was the only condition for participation. The link to the questionnaire was presented to putative respondents through social media (i.e., Twitter, Facebook, Instagram) and professional and associative mailing lists between July 2021 and October 2021. A purposive snowballing recruitment process was used to build a convenience sample with the aim of covering a broad range of sociodemographic profiles. No incentive was offered for questionnaire completion.

### Sample size

Due to the convenience sampling dissemination method of the online survey, we were unable to define the size of our source population. Based on a recent study reporting the measure of a sufficient sample for online surveys to estimate a proportion with a 95% confidence interval and 5% margin of error [27], we chose the highest estimate of 385 respondents as our sample size.

### Data analysis

All data were collected anonymously. We analyzed complete cases. Qualitative variables were described by numbers and frequencies and compared using the chi square test. Quantitative variables were expressed as the mean ± standard deviation and compared using Student’s t test. We used univariate and multivariate logistic regression models to analyze the association between self-reported social media use or expectations and several putative associated factors. Four dimensions of use or expectations were developed, namely, (1) reaching peers, (2) obtaining information, (3) seeking counseling, and (4) memorializing. The participants could report using social media for each of these dimensions. A model was built for each dimension with the same six variables identified as putative explanatory factors of use and expectations, namely, (1) age, (2) gender, (3) relationship with the deceased, (4) duration of bereavement, (5) receiving counseling, and (6) frequency of social media use. Age was dichotomized into two categories depending on the median age of the sample (more or less than 46 years old). For the relationship with the deceased, we dichotomized the category “death of a child” versus other types of relationships. The duration of bereavement was dichotomized into two categories (more or less than three years). All tests were two-tailed, and a *p* value < 0.05 was considered significant. All analyses were performed using SAS 9.2 software (SAS Institute Inc., Cary, NC, USA).

### Funding sources and ethical approval

The ESPOIR_2_S study is funded by the *Scientific Research Committee from the Centre Hospitalier le Vinatier* (funding number CSRN05) and by the *National Institute for Public Health Research* (Institut de Recherche en Santé Publique– funding number IRESP-RSP2020-230791). The study received ethical approval from the Ethical Review Board of The University Claude Bernard Lyon 1 in January 2021 (registration number 2021-01-12-04).

## Results

### Participants

The questionnaire was accessed 755 times, and a total of 401 respondents fully completed the questionnaire. Nearly 90% of the participants were women, and one-third were bereaved by the suicide of their child. A total of 45% of our sample was bereaved by the suicide of their partner, their sibling or their parent. Half of the participants had been bereaved for less than 3 years, and nearly two-thirds benefited from counseling during their bereavement process. The mean age was 45.7 years, ranging from 18 to 80. More than three-quarters of the participants had access to digital resources and used online resources daily. The characteristics of the participants are displayed in Table 1.

**Table 1:** Characteristics of the participants

	N	%
**Age (mean +/-SD, ** ***n*** ** = 399)**	45.7	(SD = 12.7)
**Gender**		
*Female*	354	88.3
*Male*	46	11.5
*Non-binary*	1	0.2
**Status of the deceased**		
*Child*	133	33.17
*Sibling*	56	13.97
*Partner*	59	14.71
*Parent*	56	13.97
*Other*	97	24.19
**Duration of bereavement**		
*> 3 years*	201	50.12
*< 3 years*	200	49.88
**Counseling**	254	63.34
Counseling by psychotherapist*	129	32.17
*Counseling by a psychologist*	99	24.69
*Counseling by a psychiatrist*	72	17.96
*Group therapy*	22	5.49
*Counseling in a charity*	71	17.71
**Frequency of Internet use**		
*Daily*	369	92.48
*Weekly*	24	6.02
*Occasionally*	6	1.50
**Frequency of social media use**		
*Daily*	308	77.58
*Weekly*	48	12.09
*Occasionally*	41	10.33
**Available digital devices**		
*Computer*	298	74.31
*Smartphone*	379	94.51
*Digital tablet*	102	25.44

*In France, psychotherapists are neither psychiatrists nor psychologists

SD = standard deviation

### Use and benefits of social media after suicide bereavement

A total of 247 participants (61.6%) reported using social media after suicide bereavement. Facebook was the most frequently used social media (91.9% of the participants), followed by WhatsApp (15.8%) and Instagram (13.0%). Nearly two-thirds of the participants (61.6%) reported their use of social media as beneficial or very beneficial for their grief process. A total of 6.6% reported their use as nonbeneficial.

#### Characteristics of the users

In the univariate analysis, the participants who used social media were significantly more frequently bereaved by the suicide of their child, bereaved for less than 3 years and receiving counseling than nonusers. Phones were the most frequent resource used to access social media. The results on the use of social media are detailed in Table 2.

**Table 2:** Characteristics of users and non-users of social media after the death by suicide of their relative

Characteristics	Users of social media	(*n* = 247)		Non-users of social media	(*n* = 145)	Test value*	DF	Significance
	n	%		n	%			p
**Age (mean– S.D.)**	45.64	(SD = 12.2)		45.69 (13.5)		0.04	397	0.97
**Gender (female)**	224	90.7		130	84.4	3.60	1	0.057
**Status of the deceased**						20.87	4	< 0.001
*Child*	100	40.5		33	21.4			
*Sibling*	30	12.1		26	16.9			
*Partner*	37	15.0		22	14.3			
*Parent*	24	9.7		32	20.8			
*Other*	56	22.7		41	26.6			
**Duration of bereavement**						28.93	1	< 0.001
*< 3 years*	150	60.7		51	33.1			
*> 3 years*	97	39.3		103	68.9			
**Counseling (yes)**	168	68.0		86	55.8	6.05	1	0.0139
**Current use of social media**						24.24	2	< 0.001
*Daily*	205	83.33		103	68.2			
*Weekly*	30	12.2		18	11.9			
*Occasionally*	11	4.5		30	19.9			
Digital resources used**								
*Computer*	113	45.7		27	17.5	33.24	1	< 0.001
*Phone*	219	88.7		41	26.6	160.15	1	< 0.001
*Tablet*	32	13.0		8	5.	-		
*Other*	0	0.0		4	2.6	-		

S.D.= standard deviation; DF = degrees of freedom

*Chi square test for all variables except age (Student’s t-test); **Digital resources types are binary variables. Chi square test was used only for computer use (yes or no) and phone use (yes or no)

#### Reasons for use

Social media were mostly used by the participants to reach peers bereaved by suicide (76.5%) and to memorialize their loved one (58.3%). Nearly half of the participants also used social media to find information on suicide and suicide bereavement (49.4%), and a minority used social media to receive or find counseling (4.0%). The results are detailed in Table 3.

**Table 3:** Reported uses of and expectations about social media in people bereaved by suicide

	Use		Expectations	
Reasons/expectations	n	%	n	%
Reaching peers	189	76.5	176	71.3
*Receiving support from peers*	91	36.8	113	45.7
*Accessing testimonies of peers*	137	55.5	144	58.3
*Chating with peers*	116	47.0	127	51.4
**Finding information**	**122**	**49.4**	**198**	**80.2**
*Finding information on suicide*	96	38.9	158	64.0
*Finding information on suicide bereavement*	102	41.3	167	67.6
*Finding were to access counseling*	15	6.1	122	49.4
**Reaching professionals**	**10**	**4.0**	**148**	**59.9**
*Receiving online counseling*	3	1.2	63	25.5
*Chating with a mental health professional*	10	4.0	145	58.7
**Memorialize his/her loved one**	**144**	**58.3**	**121**	**49.0**
*Honoring the memory of his/her relative*	144	58.3	121	49.0
*Announcing the death*	98	39.7	-	-

In the multivariate analysis (Table 4), parents bereaved by the suicide of their child were more prone to use social media to contact peers (multivariate odd ratio (OR): 5.62; 95% confidence interval (95% CI): 1.91–16.48; *p* = 0.002) and to obtain information on suicide and suicide bereavement (OR: 3.80; 95% CI: 1.40–9.64; *p* = 0.002). Participants bereaved by the suicide of their partner were more likely to use social media to find information (OR: 3.68; 95% CI: 1.61–8.94; *p* = 0.008) but were less prone to use social media to memorialize (OR: 0.24; 95% CI: 0.09–0.64; *p* = 0.005). People who used social media less frequently in their daily life were more prone to use social media after the death of their relative to find counseling support (OR: 16.27; 95% CI: 2.79–94.95; *p* = 0.002) and less likely to contact peers on social media (OR: 0.18; 95% CI: 0.04–0.75; *p* = 0.002). Younger participants were more likely to use social media to commemorate the memory of their loved one (OR: 2.06; 95% CI: 1.01–4.20; *p* = 0.046). Those bereaved for less than three years (OR: 0.43; 95% CI: 0.24–0.77; *p* = 0.005) and those bereaved by the suicide of a parent (OR: 0.32; 95% CI: 0.11–0.94; *p* = 0.038) were less prone to use social media to memorialize. No significant association was found between gender or receiving counseling and the reasons for using social media after suicide loss.

**Table 4:** Multivariate logistic regressions for factors associated with the use of social media in people bereaved by suicide

	Reaching peers (OR with 95% CI)	Obtaining information (OR with 95% CI)	Seeking counseling (OR with 95% CI)	Memorializing (OR with 95% CI)
Age (< 46yo)	1.73 (0.77–3.88)	0.70 (0.36–1.39)	0.90 (0.15–5.37)	**2.06** (1.01–4.20)*
Gender (female)	0.51 (0.15–1.77)	1.78 (0.67–4.73)	-	1.64 (0.64–4.18)
Status of the deceased (child)	**5.62** (1.91–16.48)**	**3.80** (1.61–8.94)**	4.49 (0.35–57.86)	1.41 (0.59–3.37)
Status of the deceased (partner)	2.22 (0.70–7.04)	**3.68** (1.40–9.64)**	3.50 (0.15–80.80)	**0.24** (0.09–0.64)**
Status of the deceased (parent)	0.57 (0.20–1.64)	0.58 (0.18–1.87)	-	**0.32** (0.11–0.94)*
Duration of bereavement (< 3 years)	1.48 (0.76–2.86)	1.01 (0.57–1.77)	0.64 (0.17–2.51)*	**0.43** (0.24–0.77)*
Counseling (yes)	1.92 (0.94–3.92)	0.87 (0.45–1.68)	0.79 (0.12–5.23)	0.61 (0.32–1.16)
Frequency of use (occasionally)	**0.18** (0.04–0.75)*	0.57 (0.16–2.10)	**16.27** (2.79–94.95)**	0.63 (0.18–2.27)

*p < 0.05; **p < 0.01

All models consisted of multivariate logistic regressions, with the dependent variable (1) reaching peers, (2) obtaining information, (3) seeking counseling or (4) memorializing. Each model included all the following explaining variables: gender, age (under or over 46y), relationship with the deceased (child, partner or parent vs. other), duration of bereavement (under or over 3y), receiving counseling (yes or no) and frequency of social media use (occasional, weekly or daily use); The results are given in odds ratios (OR) with 95% confidence intervals (95% CI). The odd ratio could not be measured for the association between gender, status of the deceased (parent) and seeking counselling due to the low number of responses in these subgroups

### Expectations toward online resources after suicide bereavement

The majority of participants (73.1%) considered existing online resources for suicide bereavement to be insufficient, with no significant difference between users and nonusers of social media (75.3% vs. 69.5%). Nonusers of social media were more likely than users to expect the development of a specific social media platform for people bereaved by suicide (65.6% vs. 45.4%; *p* < 0.001).

When questioned about their expectations regarding online resources after suicide bereavement, the participants who used social media reported that they mostly expected online resources to provide information on suicide and suicide bereavement (80.2%) and to facilitate access to peers bereaved by suicide (71.3%), as shown in Table 3.

In the multivariate analysis (Table 5), people who were receiving counseling were more likely to expect to access peers (OR: 2.45; 95% CI: 1.27–4.71; *p* = 0.007) and find information (OR: 2.52; 95% CI: 1.20–5.30; *p* = 0.015). Women were more prone to expect online resources to provide information (OR: 2.78; 95% CI: 1.04–7.40; *p* = 0.042), and parents bereaved by the suicide of a child expected to find counseling through online resources (OR: 2.79; 95% CI: 1.19–6.54; *p* = 0.019). Bereaved partners were less likely to expect to memorialize through social media (OR: 0.20; 95% CI: 0.07–0.55; *p* = 0.002). No differences in expectations regarding online resources were found according to age, duration of bereavement or daily use of social media.

**Table 5:** Multivariate logistic regressions for factors associated with the expectations regarding social media in people bereaved by suicide

	Reaching peers (OR with 95% CI)	Obtaining information (OR with 95% CI)	Seeking counseling (OR with 95% CI)	Memorializing (OR with 95% CI)
Age (< 46yo)	1.39 (0.66–2.89)	1.57 (0.69–3.57)	1.88 (0.93–3.78)	1.52 (0.76–3.03)
Gender (female)	1.05 (0.39–2.79)	**2.78** (1.04–7.40)*	2.08 (0.81–5.29)	1.58 (0.64–3.89)
Status of the deceased (child)	2.30 (0.94–5.63)	2.41 (0.86–6.76)	**2.79** (1.19–6.54)*	1.13 (0.49–2.59)
Status of the deceased (partner)	1.42 (0.52–3.86)	0.77 (0.25–2.37)	1.05 (0.42–2.70)	**0.20** (0.07–0.55)**
Duration of bereavement (< 3 years)	1.21 (0.67–2.20)	1.67 (0.85–3.29)	1.12 (0.64–1.94)	1.25 (0.73–2.14)
Counseling (yes)	**2.45** (1.27–4.71)**	**2.52** (1.20–5.30)*	1.80 (0.96–3.38)	1.37 (0.74–2.55)

*p < 0.05; **p < 0.01

All models consisted of multivariate logistic regressions, with the dependent variable (1) reaching peers, (2) obtaining information, (3) seeking counseling or (4) memorializing. Each model included all the following explaining variables: gender, age (under or over 46y), relationship with the deceased (child, partner or parent vs. other), duration of bereavement (under or over 3y), receiving counseling (yes or no) and frequency of social media use (occasional, weekly or daily use)

The results are given in odds ratios (OR) with 95% confidence intervals (95% CI)

## Discussion

We aimed was to evaluate the use of social media in French people bereaved by suicide and to assess their expectations toward social media through an online survey which was part of a mixed-method collaborative and participatory user-centered study that aims to build resources from the perspectives of people bereaved by suicide. We were particularly interested in assessing how social media are used by people bereaved by suicide depending on their age, gender or relationship to their loved one who has died by suicide.

First, we found a high prevalence of the use of social media in our sample of 401 people bereaved by suicide. Indeed, nearly two-thirds of the participants used social media after suicide bereavement, and the vast majority of them used Facebook. This result is consistent with the common idea that social media has an important place in contemporary grief processes. Dilmaç [28], for example, emphasized that the digital age is modifying three primary characteristics of death. First, the chronological aspect of death implies that there is a before and an after death, although the deceased can live a second life on the internet. Second, death implies a physical separation between those who are alive and dead persons who are no longer part of the world, but no such separation exists in the virtual world. Third, death rites are needed so that the departed and their loved ones can gain slightly greater acceptance of the fatal truth. In this sense, funeral rites are characterized by their institutionality because they concern not only the individual but also the community and its culture [29]. Again, social media do provide “new forms of mourning”, described as a new funeral rituality [29, 30] that is specific to the Web and signifies “a complexification of our relationship to death” [31]. Our results highlight that social media can contribute to grief processes, such as sense- and meaning-making, by allowing people to access information on suicide or discuss their feelings or emotions with peers.

In our sample, parents bereaved by the suicide of their child were significantly more likely to use social media than others and tended to report specific patterns of use and expectations regarding social media for their grief process. This population is among the most heavily impacted groups after suicide loss [32] and displays high needs in the aftermath of the suicide loss. Notably, they might be more likely to expect to receive support messages on social media compared to other members of the family to cope with the feelings of shock, bewilderment and emptiness caused by the loss of their child. In a longitudinal qualitative study, Entili et al. [33] reported respondents’ changing needs and coping strategies over two years, with differentiation between strategies adopted by fathers and mothers. According to our results, social media could play an important role in supporting bereaved parents by helping them contact peers and obtain information on suicide and suicide bereavement.

Interestingly, we found that younger participants of our sample were more prone to use social media to memorialize than older participants. This result would be consistent with the hypothesis that social media is modifying the way new generations are mourning and commemorating the memory of their loved ones. Recently, King & Carter [34] performed a qualitative study of fourteen bereaved young millennials. The authors found that the participants gave great importance to social media for mourning. Notably, continuing bonds and expressing feelings and emotions were reported as the main motivations to use social media for grieving. Many participants in the study of King and Carter explained that they posted pictures, memories, videos and statements to continue their bonds with the deceased. Moreover, the participants explained that they posted directly to the deceased on birthdays and death anniversaries. Two participants bereaved by suicide in their study also noted that the reason they posted their grief online was to reduce the negative stigma attached to suicide.

According to our results, people in our sample who were recently bereaved were more likely to use social media than those who had been bereaved for more than three years. Further studies should examine how social media are used throughout the years during the grief process of people bereaved by suicide. Contemporary theories of grief describe it as a dynamic process in which the immediate aftermath is the most difficult stage, but periods of increased grief are found at points in the assimilation trajectory. It is possible that the suicide risk may be elevated at any of these points, e.g., in the immediate aftermath of the death and perhaps on anniversaries of the death. Anniversary reactions are conceptualized as psychological, somatic and behavioral reactions to temporal triggers of an anniversary of a substantial event [35]. There is some evidence to support this hypothesis, and several studies have reported an increased suicide risk and impaired mental health on bereavement anniversaries [36–40], while a recent study did not find any evidence of an elevated suicide risk around anniversaries in people bereaved by suicide [41]. Digital resources, such as memorial-based websites, may offer new ways to contact and support bereaved people [16, 17] and to implement innovative and adaptive prevention strategies to reduce the deleterious effects of bereavement anniversaries on mental health.

Unexpectedly, no differences were found between men and women according to their use and expectations of social media. In contrast, previous studies reported that men were at higher risk of not obtaining support after the suicide of a loved one and that men displayed specific patterns of grief [33, 42, 43]. This could mean that social media may constitute an interesting means to facilitate access to help and support for bereaved people who are usually lacking support. However, this result could also be explained by a lack of power due to the small number of male participants in our sample (11.5% of the sample).

### Implications

According to our results, the role of social media in people bereaved by suicide is important, especially in the early period of bereavement. Based on our results and a recent expert consensus on postvention interventions [44], several recommendations can be provided for the use of social media to support people bereaved by suicide. First, social media must be considered a critical means to support people bereaved by suicide as an addition to, rather than a substitute for, other sources of support, such as face-to-face counseling or peer support groups. Second, social media should be seen as a way to disseminate information on suicide bereavement and to help people bereaved by suicide contact peers. Third, the special needs of some categories of people bereaved by suicide (especially parents bereaved by the suicide of a child or youth) must be met by social media. Fourth, evidence-based interventions using social media must be evaluated to better understand how they can help people bereaved by suicide in bereavement processes such as memorialization and sense- and meaning-making. Fifth, there is an urgent need for active engagement between mental health professionals and the social media industry to design appropriate and ethical social media platforms dedicated to supporting bereaved individuals. The community of those affected by mental health and suicide bereavement could indeed utilize social media to raise awareness and encourage individuals to seek or offer help in cases of mental distress.

### Strengths and limitations

We performed the largest online survey on the use and expectations of people bereaved by suicide with regard to social media. With a total of 401 participants, we exceeded the goal of 385 participants for a convenience sample survey [27]. However, our study has several limitations. First, we performed a cross-sectional study, so no causal inference can be made based on our results. Second, the majority of participants were women, which may impede the generalizability of our results. However, the ratio was consistent with the ratios of previous studies on suicide bereavement [16, 45–47]. This may be explained by a higher propensity for bereaved women to participate in research compared to bereaved men [45, 47] and by the higher prevalence of suicide bereavement in women compared to men [48]. Third, we included only adults, so we could not collect data on the use of social media by bereaved children or adolescents. However, children and adolescents use social media with potential risks for mental health [49]. Further studies should assess how children and adolescents bereaved by suicide use social media. Fourth, our study was performed in France, so our results and conclusions must be generalized with caution. Finally, the use of an online survey may have induced selection bias because people who do not often use online resources may have been less prone to participate in our study. It is notable that the vast majority of the participants reported daily use of the internet and social media. However, the Internet has been identified as a compelling means to recruit people bereaved by suicide to participate in research [47].

## Conclusion

We performed a large study assessing the use of social media after suicide bereavement in a French sample of bereaved adults. A large part of people bereaved by suicide use social media for their grief process, mainly to contact peers bereaved by suicide and to memorialize their loved one. Younger participants are more prone to use social media to memorialize, indicating that social media is modifying the way new generations are mourning and commemorating the memory of their loved ones. Thus, social media contributes to contemporary grief processes and can be seen as a putative means to improve the mental health of people bereaved by suicide.

### Electronic supplementary material

Below is the link to the electronic supplementary material.

Supplementary Material 1

## Data Availability

The datasets used and/or analyzed during the current study are available from the corresponding author, EL, on reasonable request.
